# Analgesic use by ageing and elderly patients with chronic non-malignant pain: a qualitative study

**DOI:** 10.1007/s11096-017-0466-y

**Published:** 2017-05-04

**Authors:** Mary-Claire Kennedy, Grainne Cousins, Martin C. Henman

**Affiliations:** 1 School of Healthcare, Baines Wing, University of Leeds, Leeds, LS29JT UK; 2 School of Pharmacy and Pharmaceutical Sciences, Trinity College Dublin, Dublin, Ireland; 3 School of Pharmacy, Royal College of Surgeons in Ireland, St. Stephens Green, Dublin, Ireland

**Keywords:** Ageing, Analgesics, Chronic treatment, Health behavior, Ireland, Medication, Pain, Qualitativce research

## Abstract

*Background* Analgesics are used in the management of chronic non-malignant pain (CNMP), a condition which is highly prevalent among older adults. CNMP may not only be physically distressing but also complicated by psychosocial and economic factors. An individual’s perception and use of analgesics may be influenced by a range of factors such as perceptions of risk or benefits, ability to purchase medication or access to non-pharmacological therapies or specialist care. *Objective* The aim of this study was to describe the perceptions and experiences of analgesics by ageing and elderly individuals with CNMP and identify factors that influence their use. *Setting* Telephone interviews with 28 members of Chronic Pain Ireland aged ≥50. *Method* In-depth semi-structured interviews; audio-recorded, transcribed verbatim, and thematically analysed. *Main outcome measure* Experiences and perceptions of ageing and elderly individuals with CNMP taking analgesics. *Results* A combination of factors specific to the patient and arising from outside influences informed perceptions and experiences of analgesics. Pain severity, perceived efficacy of analgesics, occurrence of adverse-effects and concerns about addiction/dependence were identified as internal factors influencing medication use. External factors included views of family members, access to specialised care and the individual’s interaction with healthcare professionals (HCPs). *Conclusion* Individuals with CNMP regard analgesics as an important method for managing pain and are relied upon when other interventions are difficult to access. HCPs in primary care, who are the main point of contact for patients, need to take into account the various factors that may influence analgesic use when consulting with this patient group.

## Impacts on practice

- While analgesics are an important method of pain control, patients attempt to offset use through nonpharmacological management strategies, but limited access to physical and psychological interventions contributes to increased reliance on analgesics for pain management.
- Primary care physicians must be offered opportunities to engage in continuing professional education on the subject of analgesia as new therapies are licensed and concerns about existing medications emerge.
- Patients have concerns about long-term use of strong opioids which is perceived to be inherently linked with addiction. Before starting treatment, these concerns should be discussed with patients who need strong pain-control.

## Introduction

Chronic non-malignant pain (CNMP) is defined as pain that exists beyond 3 months or the expected timeframe for healing [1]. It is prevalent among ageing and elderly patients due to the development and progression of chronic degenerative conditions [2]. The burden of CNMP is certain to increase in future years with our globally ageing population [2, 3].

Analgesics such as non-opioids [e.g. paracetamol and non-steroidal anti-inflammatories (NSAIDs)] or opioids are commonly used to manage CNMP. A recent European survey suggested that two-thirds of individuals with CNMP were taking some form of analgesic [4, 5]. Depending on the nature of the pain condition adjuvants may be used, examples include anticonvulsants, antidepressants and muscle relaxants [6]. In common with all medications, optimal outcomes are achieved when analgesics and adjuvants are taken as prescribed, the clinician having considered the risks and benefits of the medication for that patient. Elderly patients are at increased risk of harmful outcomes from medications due to physiological changes that alter pharmacokinetics and pharmacodynamics in addition to multi-morbidities and polypharmacy [7, 8]. These challenges limit the range of pharmacotherapeutic options available to the clinician.

The pattern and extent of analgesic use is also influenced by the patient’s perceived need for, efficacy of, and concerns about the medication, some of these concerns particular to older patients [9, 10]. Stoicism of elderly patients may lead to an increased tolerance towards painful conditions and a tendency to take analgesics less frequently or at a lower dose than prescribed [11]. Additional factors affecting use of analgesics by patients with CNMP have been described in the literature including addiction concerns, unfavourable scrutiny, adverse effects, tolerance, withdrawal and mistrust in the prescriber [12]. Despite these issues, there is a steady increase in the use of prescription opioids in several countries including the USA, UK, Germany, Australia and Canada [13–17]. In recent years, there has also been an increase in the prescribing of gabapentin and pregabalin with a simultaneous rise in reports of misuse of these medications [18–20]. However, few studies have considered attitudes of patients towards these adjuvants, particularly in light of this emerging information on misuse of these agents.

The NICE Medicines Adherence guideline (2009) details recommendations for the involvement of patients in decisions about prescription medications [21]. The guideline references Horne’s model of adherence as a framework to consider the various factors that may affect the manner in which patients take their medications [22]. The model, which builds upon the self-regulatory model of illness, describes internal factors including beliefs about disease and medications, the perception of the role of medications in disease management, and the patient’s ability to take medications [22, 23]. External factors include the sources from which patients gain information about medications including healthcare professionals (HCPs), family, friends and media, the financial implications of medication use and the influence of regional or national health policy [21, 22]. Using this framework to consider the factors affecting medication use by patients with CNMP is of particular relevance to HCPs seeking to implement NICE recommendations [21, 24].

## Aim of the study

This study aimed to examine the experiences of ageing and elderly individuals with CNMP with analgesics, and to describe the factors that influence the way these medications are used by this group.

## Ethics approval

Ethical approval was granted by the Health Sciences Research Ethics Committee, Trinity College Dublin (TCD).

## Methods

Semi-structured telephone interviews were conducted with members of Chronic Pain Ireland (CPI), a patient support organisation with approximately 300 members. An e-mail invitation was sent to all members and an advertisement was placed both on the CPI’s Facebook^®^ and Twitter^®^ pages and in the quarterly newsletter. Individuals aged >=50 years were eligible to partake in this study. Individuals of this age are classified as ‘ageing’, as they may have retired, are no longer of child-bearing potential and will be considered elderly within a number of years [25]. It is important that economic, social and healthcare policy planners are aware of the needs of this group before reaching old age. For these reasons, longitudinal studies on ageing conducted internationally have included individuals aged 50 and older [26].

Individuals could not take part in the study if they had a terminal illness or had been diagnosed with dementia or any other memory impairment. Any member of the CPI who volunteered to participate and were eligible for inclusion were sent an interview invitation. Participants were allowed at least 5 days between signing the consent form and conducting the interview.

A pragmatic approach was adopted to this study with the focus on identifying themes rooted within the data to capture participants viewpoints [27]. Data was managed using the framework method and analysed through applied thematic analysis, which can be viewed as a methodology in its own right [28, 29]. An interview guide was developed by the research team, questions were constructed by reviewing the literature and professional experience. The guide was piloted with a qualitative researcher who recommended revisions based on their experience with semi-structured interviews. The interview guide is outlined in “Appendix 1”, it includes probing statements that were used to obtain some detailed information or to follow up on points of interest. All interviews were conducted by telephone by MCK who had training and experience in qualitative interviews. Interviews were recorded using the Audacity^®^ software package and a portable recording device. The research team monitored the thematic content as the interviews progressed, as no new themes emerged at the 24th interview, data saturation had been reached and only the interviews that had already been arranged were completed [30]. Transcripts were reviewed by participants and were then transferred to NVivo^®^ Version 10. A member of the research team (MH) reviewed a sample of the transcripts to ensure that coding and thematic analysis were exhaustive.

## Results

Twenty-eight interviews were conducted over 6 months (May–October 2013). Twenty-seven transcripts were retained for analysis as 1 participant did not wish to proceed with the study after reviewing the transcript. Interviews were an average of 36 min. Nine men and 18 women participated in the study and were aged 50–70 years. “Appendix 2” details the age ranges, pain conditions and current classes of pain medications of participants.

The Horne Model applied after completing thematic analysis allowed for the identification of internal factors affecting analgesic use which included pain severity and the perceived efficacy of analgesics, concerns and negative experiences with medications and engagement in self-education or use of non-pharmacological interventions. External factors included influences of family and friends, access to specialised care and interaction with HCPs [21].

## Internal factors

### Pain severity and analgesic efficacy

Pain affected participants in different ways; some had limited ability to undertake daily activities such a washing and dressing, while others were limited only when they sought to participate in additional activities such as walking distances or driving. All participants contrasted previous activity levels with current abilities to contextualise pain severity or to describe disease progression.

That is the other thing; my stamina isn’t nearly what it was. I could do a full day’s work and do three meetings back to back whereas if I can do one expedition out of the house now in one day it is about as much as I can manage. (*P28, female, chronic back pain*)

Participants discussed their perception of the risks and benefits of analgesics by describing the efficacy of the medication in delivering pain relief relative to the negative effects of the medication. Embedded within these discussions regarding efficacy weighed against risk was a sense of acceptance of the unremitting nature of the condition and the necessity to take analgesics continuously. All participants indicated that analgesics were an important aspect of their approach to pain management, enabling them to undertake daily activities. In cases where medications provided effective analgesia, participants described the importance of adherence to the prescribed regimen as pain may recur upon omission of a dose:

If I happen to forget taking them or maybe went off by mistake, and I wouldn’t have taken them, I’d know that evening that it would be much, much more painful. (*P8, male, peripheral neuropathic pain*)

My perception of it is that it’s essential for the maintenance of my health and wellbeing. It also assists in maintaining my relationship with my wife, my children and my grandchildren. (*P13, male, rheumatic condition*)

However, this is contrasted with negative perceptions of analgesics voiced by a number of participants who viewed them as perpetuating a negative cycle of use which they are unable to stop:

…it is a terrible vicious circle that you get into, you are taking the medication, you know it is not going to cure it and at the same time you keep taking it in the hope of just bringing it down a few levels [pause] … I think if I was dealing with somebody else, I would be telling them for God’s sake [sic] come off that medication, and here I am. I am caught up in it. (*P18, female, chronic neuropathic pain*)

### Adverse effects

Adverse effects were described for NSAIDs, opioids and adjuvants however, those arising from opioids appeared to cause most distress. Participants appeared to be aware of the adverse gastrointestinal (GIT) effects of NSAIDs, however, no one discussed the potential adverse cardiovascular effects. One participant described the development of a stomach ulcer which she stated was caused by diclofenac:

I have osteoarthritis, a pain in my back, then eventually I got a stomach ulcer from taking Difene^®^ [diclofenac]. They say that it may have been stress but it may have been Difene^®^ (*P27, female, arthritic pain*)

Adjuvants including pregabalin, gabapentin, amitriptyline and duloxetine were described as causing adverse effects among participants which in some necessitated stopping the medication:

The other side effect … is that I am overweight, and that is because of the increased appetite or things like that things, cravings for sweet foods because of medication, again that is to do with side effects. (*P9, female, rheumatic condition*)

I was told to try amitriptyline … now that [pause] even the smallest amount of that would have me asleep for 24 h so I decided not to. (*P10, female, rheumatic condition*)

The adverse effects of strong opioids which caused greatest distress were GIT upset and sedation. For some participants, these adverse effects necessitated discontinuation of the medication, even if this medication was effective in providing pain relief. One participant also described the sense of detachment associated with opioids as impacting on her ability to function on a daily basis:

That would have been the main reason I would have taken a break from them. I found that I was too sedated [pause] not so much sedated more that I felt maybe a detached, like feeling like the world was going on around me. Even the children were saying that my favourite hobby was sleeping! (*P26, female, rheumatic condition*)

### Addiction and tolerance

The potential for substance dependence with opioids is widely known and participants often voiced such concerns during the interviews. Notably none of the participants taking adjuvants such as pregabalin and gabapentin expressed similar concerns about these medications. There were, however, differing degrees of concern about the potential for developing addiction or tolerance to opioids:

What I have learned is the opiates are only [pause] I understand people take them for a long time but there should be something that stops you, because you eventually reach the stage where you need more and more to get the same effect. (*P23, male, chronic neck pain*)

Addiction would be of a concern for me and with the family here and you’re concerned about things like this. And they are very much aware of what they do. And they’re concerned that I might be dependent too much on them. (*P16, male, central neuropathic pain*)

### Long-term health

Participants discussed the long-term health implications and potential problems of taking analgesics. While there was a sense acceptance among participants that analgesic use will continue indefinitely with a likely increase in the quantity and dose of medication, this raised concerns about the impact of medication on their future physical health, tolerance to the medication and lack of treatment options:

It was going to relieve me of my pain, but really the end result would be that it would damage my system. (*P24, female, peripheral neuropathic pain*)

I was speaking to a colleague at work about the very same thing, we were sitting on night duty talking about it and she said, now you might die younger, but at least you won’t be in as much pain. [Laughter] (*P21, male, chronic back pain*)

### Self-education and non-pharmacological interventions

Participants referenced access to and use of patient support groups and have incorporated strategies discussed in workshops and meetings into their every-day routine. Several participants who had attended a pain management programme outlined the improvements the programme had made to management of their condition:

Now one thing I was doing which was wrong, I was watching the clock and saying, oh I can take another tramadol, you know what I mean, I’d say every 4 h. [pause] With the pain programme I stopped doing that. (*P14, female, arthritic pain*)

Participants also discussed their use of physiotherapy, hydrotherapy, acupuncture, pilates, yoga, cognitive behavioural therapy and transcutaneous electrical stimulation (TENS). Some were using one or more of these interventions to minimise or offset the need for an analgesic:

I’d normally start with one [tramadol tablets] and hope it works, but I do usually end up having to take a second one [tramadol tablets] and I kind of, try and use heat or a TENS machine or something else rather than take anymore (*P7, female, chronic head/pelvic pain*)

Several participants described accessing information on the internet and highlighted the contribution of this information towards their personal appraisal of their medication regimen or their consultation with the prescribing physician:

If I go to somebody and they prescribe something, the first thing I do is go to the internet and look it up. And, what tends to be on the internet…is quite a lot of negative stuff. And again you’re starting off with a negative base… (*P3, male, chronic back pain*)

## External factors

### Third party influences

Participants have developed an understanding or perception of their condition through personal experience and interaction with family members, friends and other patients. Family members were universally concerned about the pain experienced by participants and were perceived to be more accepting or encouraging of analgesic use than others. The support received from family members appears to dispel some of the reservations participants may have about taking analgesics. This is in stark contrast to the sense of stigmatisation and misunderstanding that individuals perceived with the general public.

Probably my family would be different because they’ve actually seen me and seen me in pain and they would be very much inclined to say look you know you’re going to end up taking it, why don’t you just take it now instead of in 4 h time when you’re much worse. They would try to encourage me to take it much more. (*P7, female, chronic head/pelvic pain*)

I suppose what I would find about the community in general is that nobody understands the word chronic… So when people see you and you are looking well and tanned… you must be better. (*P18, female, chronic neuropathic pain*)

### Healthcare professionals

All participants discussed the care received from their GP, with the majority speaking positively about this experience. Participants considered the clinical knowledge and the personal experience of interacting with their GP as the most important topics when discussing GP care. While some participants were satisfied with the management received, some did express the opinion that GPs had not received adequate specialised training on CNMP:

The GPs, there is a real need for more training and up-skilling in the area, with me they just don’t know what to do anymore and they haven’t known what to do for a good while really. (*P12, female, pelvic pain*)

Despite the perceived limitations of GP’s clinical knowledge of CNMP, participants spoke warmly of these relationships. They understood that GPs wished to help and often independently and proactively researched the condition to aid their patient. Several participants reported that they also relied on the GP to ensure that the care received from the various HCPs was harmonised in primary care:

My doctor… she is excellent… and she kept going until she got a diagnosis, and she is proactive in looking up new medication and trying to get it right, you know, get it sorted, and as I said I always get her to check what the hospital say. (*P2, male, chronic back pain*)

### Specialist care

Participants referred to the financial and geographical restrictions affecting access to specialised care. Financial barriers related primarily to the inequity of the current public–private structure of the Irish healthcare system. Health insurance or private payments enabled patients to bypass public waiting lists, gaining faster access to consultant level care, however this placed considerable financial burden on most participants. The cost of healthcare, particularly medications and private specialist care, was a major concern particularly for those seeking healthcare privately. This is contrasted with difficulties associated with access to similar services in the public system. Participants appear to factor in the cost of management of their condition as a contributory barrier to effective management:

The way I have sort of gone paying, not that I can afford it, but paying you will get in somewhere much quicker anyway and you sort of have to weigh up things. (*P1, female, rheumatic condition*)

Participants were asked to comment on their experience of interdisciplinary co-operation in the management of their condition. While participants tended to praise the care received in primary care, and acknowledged the expertise of specialists, the connectivity of these domains clearly remains an issue:

I know there were doctors who were writing out things but no one was paying any attention to what the other fella [sic][*meaning: man*] was doing. (*P23, male, chronic head/ neck pain*)

## Discussion

This study highlights the multiple complex factors that can influence the use of analgesics by ageing and elderly individuals with CNMP. These factors can be mapped onto those identified within Horne’s model of adherence, this framework is of particular importance to clinicians seeking to optimise analgesic use among patients with CNMP in accordance with the NICE Medicines Adherence guideline (2009) [21, 24].

Ability to undertake daily activities or engage in social or leisure pursuits appears to be an implicit measure employed by patients to establish the efficacy of the analgesic regimen [31]. However, these perceived benefits of both analgesics and adjuvants are balanced with the risks associated with the medications. There is extensive literature on the clinical complexities of prescribing medications for ageing and elderly patients and it would appear that this group were somewhat aware of these potential problems citing specific issues such as adverse effects and drug interactions. Adverse effects appear to be the most significant influence on analgesic use or non-adherence to the prescribed regimen. This finding is similar to that of quantitative studies of opioid and NSAID adherence which have indicated that adverse effects such as GIT effects or sedation contribute to cessation of these medications [32, 33]. Addiction or dependence arising from the use of strong opioids did not appear to concern participants to the same extent as other adverse outcomes. Although concerns were vocalised by a few participants, this was balanced with an acceptance of the role of strong opioids in pain management and the potential that these medications might be used on a long-term basis. This is at odds with numerous studies with both patients and physicians which detail concerns about opioid related aberrant behaviours and the development of dependence or tolerance [11, 12, 34–38]. In addition, the potential for misuse of adjuvants was not addressed by any participant. It is probable that this is due to widespread public knowledge of the negative aspects of strong opioids which is not associated with other drug classes at present.

External factors can exert effects on the management of the patient with CNMP. HCPs and social support networks can influence the patient’s approach to coping with the condition [39]. Positive interactions with these networks can reduce stigma and encourage acceptance of both the chronic pain condition and the necessity for long-term use of analgesics and perhaps help to minimise stoicism of this patient group reported in the literature [40, 41]. HCPs, particularly those responsible for prescribing medications, contribute to a patient’s understanding and acceptance of analgesics. A positive patient-physician relationship has been demonstrated to reduce patient concerns and negative perceptions of medication [42, 43]. This study has highlighted the supportive relationships between the participants with their GPs and is contrary to that of similar qualitative studies in which patients with CNMP have described the feeling of being a burden to the GP or viewed as a drug seeker by HCPs [44–47]. The shared decision making with GPs described by participants is further evidence of this supportive relationship. This is associated with enhanced adherence to the medication regimen, with patients also reporting a greater reduction in pain than those managed through the paternalistic model [48].

Several participants in this study described a concern that GPs lack sufficient knowledge on CNMP to provide effective management in primary care. This concern is echoed in empirical studies which have highlighted insufficient focus on pain education within undergraduate medicine curricula leading to a lack of confidence when managing patients with CNMP [49–51]. Engagement with clinicians is further hampered by ineffective communication and co-ordination between primary care and specialists as well as the difficulties in accessing specialist care. The financial and geographical barriers to access were referenced by all patients receiving care through the public system, of particular significance to ageing and elderly patients, the majority of whom are dependent on public healthcare [52]. Furthermore, negative experiences in specialist care or suboptimal analgesia achieved by clinical interventions in this setting may cause the patient to return to primary care seeking further interventions. This desire to establish relationship continuity with a HCP has been reported to be of high importance to older patients and those with chronic conditions [53]. This study suggests that a HCP with specialised clinical knowledge of CNMP, who is easy to access and possesses good knowledge of the patient’s medical history would be best placed to co-ordinate and optimise management.

Patients are becoming an increasingly empowered group with access to information independent of HCPs, mainly via the internet [54]. Participants discussed their efforts to self-educate together with incorporating non-pharmacological interventions which supplemented or offset analgesic use. These measures indicate a willingness to self-manage the condition, a behaviour associated with positive health outcomes for CNMP including fewer GP and hospital visits and improved health behaviours [55, 56]. Previous studies have highlighted variability in the extent to which older patients are prepared to participate in self-management [57]. In essence, a willingness to self-manage the condition may be an effort to self-regulate use of analgesics, particularly those medications that patients view in a negative light such as opioids; this theory of self-regulating use of strong opioids has recently been reported by Paterson et al. [58]. It should be considered that engaging in self-management behaviours with a view to offsetting medication use, as identified in this study, is a potential barrier to optimising pain relief for the patient.

There are several limitations to this study. Participants were members of CPI and self-selected into the study which demonstrates a degree of self-empowerment and interest in their condition. Such self-empowerment may not be representative of the general ageing and elderly population with CNMP [59]. Interviews were conducted by telephone as this is an inexpensive, efficient method for interviewing a relatively large number of participants [60, 61]. Telephone interviews also allowed individuals to participate from a location which they could arrange to suit their physical needs. The main disadvantage is the loss of nonverbal cues gained from face-to-face interaction and the opportunity to use visible aids during the discussion. Finally, analysis of qualitative data may introduce bias into the study as the researcher may approach the topic with pre-formed opinions and ideas. We have sought to address this through extensive documentation, auditing and recording of recruitment and analytical strategies allowing external parties to review the reflexivity of the research process [62].

## Conclusion

This study described the factors affecting the use of analgesics by ageing and elderly adults with CNMP. Internal factors including efficacy of the analgesic together with the perceived risks of the medication are specific to the patient and influence the nature and extent of analgesic use. External factors, such as poor access to pain specialists, physiotherapists or other allied healthcare professionals, affect the quality of management of the condition. Awareness of these factors is of value to HCPs when seeking to optimise analgesic use within this patient group.

## Appendix 1

**Table:**

Question theme	Statements/questions in interview guide
Introduction and information for participants	Describes the aims and objectives of the study
Demographic information and background on pain condition	Can you tell me what is the cause of your chronic pain?
	Duration of pain?
	Pain intensity/severity?
	Impact of pain on activities of daily living?
Impact of pain and pain medications	Would you be able to name the painkillers that you take for your pain? (*Prompt frequency*)
	Can you describe when you take prescribed pain medications (triggers)?
	Are there certain times when you take more painkillers than others?
	Do you get satisfactory pain relief from the prescribed pain medications you are currently taking?
	Have you ever avoided taking prescribed painkillers? (*Prompts*-*no perceived benefit/cost/side*-*effects/overuse*) (*if necessary follow*-*up*)
	Do you ever increase/reduce the number of tablets you take or avoid taking medication?
Management of analgesic regimen	Have you ever stopped taking a painkiller prescribed for you? What was the reason?
	Do you forget to the pain medications prescribed for you?
	Do you use any tools to help you remember to take your medications e.g. pill boxes, diary?
Perceived barriers to management of CNMP with analgesics	Do you feel that you can discuss your pain medications with your doctor?
	Do you think there is more negativity surrounding the use of painkillers than other medications?
	Do you think that pain medications are more addictive or equally as addictive when compared with other types of pain medications?
	Did you try these treatments in addition to medications or as an alternative to medications?
Supports available to patients in the healthcare system	What is your opinion of the support available to chronic pain patients in the management of their condition for instance your access to:
	Physiotherapists
	Occupational therapists
	Pain clinics and specialists?

## Appendix 2

**Table:**

Participant	Gender	Age group	Years with CNMP	CNMP condition	Class of medication
1	F	60–70	<5	Rheumatic condition	NSAID (topical), non-selective NSAID
2	M	50–59	11–20	Chronic back pain	Anti-epileptic, non-selective NSAID
3	M	50–59	5–10	Chronic back pain	Paracetamol/weak opioid
4	F	50–59	>20	Chronic back pain	Paracetamol/weak opioid, non-selective NSAID
5	F	50–59	5–10	Pelvic pain	Anti-epileptic, anti-depressant, paracetamol/weak opioid
6	M	50–59	11–20	Chronic back pain	Weak/moderate opioid
7	F	50–59	>20	Chronic head/pelvic pain	Paracetamol/weak opioid, non-selective NSAID/proton-pump inhibitor, selective 5-HT1 agonist
8	M	>50 (age not declared)	5–10	Peripheral neuropathic pain	Anti-epileptic, weak/moderate opioid, paracetamol, anti-depressant
9	F	>50 (age not declared)	<5	Rheumatic condition	Anti-depressant, anti-epileptic, weak/moderate opioid
10	F	50–59	<5	Rheumatic condition	Anti-epileptic, strong opioid, non-selective NSAID
11	F	50–59	5–10	Central neuropathic pain	Anti-epileptic, paracetamol, muscle relaxant
12	F	>50 (age not declared)	5–10	Pelvic pain	Anti-depressant, paracetamol, non-selective NSAID
13	M	60–70	11–20	Rheumatic condition	Strong opioid (patch), local anaesthetic (patch), weak/moderate opioid, paracetamol, non-selective NSAID (oral), muscle relaxant(× 2), anti-depressant
14	F	50–59	5–10	Arthritic pain	Anti-epileptic, weak/moderate, paracetamol, local anaesthetic (patch)
15	F	60–70	11–20	Chronic back pain	Strong opioid, selective NSAID (oral), weak opioid, paracetamol/weak opioid, non-selective NSAID (topical), muscle relaxant
16	M	60–70	>20	Central neuropathic pain	Strong opioid (patch), strong opioid, non-selective NSAID (topical), paracetamol
17	F	50–59	<5	Rheumatic condition	Anti-depressant, paracetamol
18	F	50–59	11–20	Peripheral neuropathic pain	Weak/moderate opioid, strong opioid, anti-epileptic
19	F	50–59	11–20	Chronic back pain	Weak opioid, paracetamol
20	F	50–59	<5	Arthritic pain, rheumatic condition	Anti-epileptic, non-selective NSAID (oral and topical), paracetamol
21	M	50–59	11–20	Chronic back pain	Paracetamol/weak opioid, anti-epileptic, anti-depressant, non-selective NSAID (oral)
22	M	60–70	5–10	Peripheral neuropathic pain	Weak/moderate opioid/paracetamol, anti-depressant, anti-epileptic
23	M	50–59	5–10	Chronic head/ neck pain	Strong opioid/antagonist, paracetamol
24	F	60–70	5–10	Peripheral neuropathic pain	Paracetamol/weak opioid
26	F	50–59	5–10	Rheumatic condition	Strong opioid (patch), paracetamol/weak opioid
27	F	50–59	<5	Arthritic pain	Paracetamol, strong opioid, non-selective NSAID (topical)
28	F	60–70	>20	Chronic back pain	Weak/moderate opioid/paracetamol, non-opioid, paracetamol (prn)
